# White Matter Stroke Masquerading as Subarachnoid Hemorrhage After High-Risk Percutaneous Coronary Intervention: A Case Report

**DOI:** 10.7759/cureus.45632

**Published:** 2023-09-20

**Authors:** Adnan Shah, Sanila Mughal, Usha Kumari, Salim Surani, Muneeb Jan

**Affiliations:** 1 Cardiology, Lady Reading Hospital, Peshawar, PAK; 2 Medicine, Dow University of Health Sciences, Karachi, PAK; 3 Anesthesiology, Mayo Clinic, Rochester, USA; 4 Medicine, Texas A&M University, College Station, USA; 5 Medicine, University of North Texas, Dallas, USA; 6 Internal Medicine, Pulmonary Associates, Corpus Christi, USA; 7 Clinical Medicine, University of Houston, Houston, USA; 8 Internal Medicine, Lady Reading Hospital, Peshawar, PAK; 9 Cardiology, Rehman Medical Institute, Peshawar, PAK

**Keywords:** white matter lesions, left main stem occlusion, coronary artery disease (cad), sah- subarachnoid hemorrhage, ischemic stroke, primary percutaneous coronary intervention (pci)

## Abstract

Considering the context of percutaneous coronary artery angiography (PCI), stroke is a rare but severe complication and is associated with high morbidity and mortality. A computed tomography (CT) scan of the brain is an indispensable imaging modality to diagnose ischemic stroke changes following PCI. A 75-year-old female who presented with sudden onset chest pain was diagnosed with anterior-wall myocardial infarction which required primary PCI. However, an hour following the procedure, she suddenly developed drowsiness, confusion, and hemiparesis. Non-contrast CT showed hyperdense signals in posterior falx and tentorium cerebelli suggesting subarachnoid hemorrhage (SAH) as well as low attenuation signals in bilateral periventricular region suggestive of microvascular ischemic changes. It was critical to decide about the continuation of dual antiplatelet therapy (DAPT), aspirin and P2Y12 inhibitor, as soon as possible. Based on the clinical presentation and mixed picture on the CT scan, a second opinion was sought by a multidisciplinary team, which concluded that the findings were consistent with white matter stroke and DAPT was resumed. The hemiparesis improved gradually with the reversal of CT scan findings. There is a lack of reported literature about ischemic stroke and SAH following high-risk PCI and what should be the best approach in ambiguous cases. The management of white matter stroke and SAH is contrasting, particularly in deciding whether to continue the DAPT after PCI; hence it is critical to diagnose them promptly. Thus, this case highlights the importance of differentiating SAH from white matter stroke for prompt treatment of post-PCI complications to ensure positive outcomes.

## Introduction

One of the most popular methods of coronary revascularization for patients with acute coronary syndrome and stable ischemic heart disease is percutaneous coronary intervention (PCI) with stent implants, particularly drug-eluting stents [1]. Stroke is an uncommon but devastating consequence of high-risk PCI procedures, associated with high morbidity and mortality. The diagnosis of an ischemic stroke after PCI requires the use of a brain computed tomography (CT) scan. However, it has been noted that contrast-enhanced CT scans and brain magnetic resonance imaging (MRI) increase the risk of contrast-induced neurological injury (CINI), particularly in patients with renal failure [2,3]. Here we describe a case of an elderly female who developed hemiparesis, drowsiness, and confusion an hour after high-risk PCI, and her CT brain findings were consistent with both subarachnoid hemorrhage (SAH) and ischemic stroke. It is critical to diagnose such cases accurately and timely since the decision to continue the dual antiplatelet therapy (DAPT) would be a matter of life and death. Based on the multidisciplinary opinion, we diagnosed her with post-PCI white matter infarction. She continued to receive DAPT and her symptoms of stroke improved over time. This case highlights the importance of accurately and efficiently differentiating the complications of PCI such as SAH and ischemic stroke and managing it in the best interest of the patient.

## Case presentation

**Figure 1:**
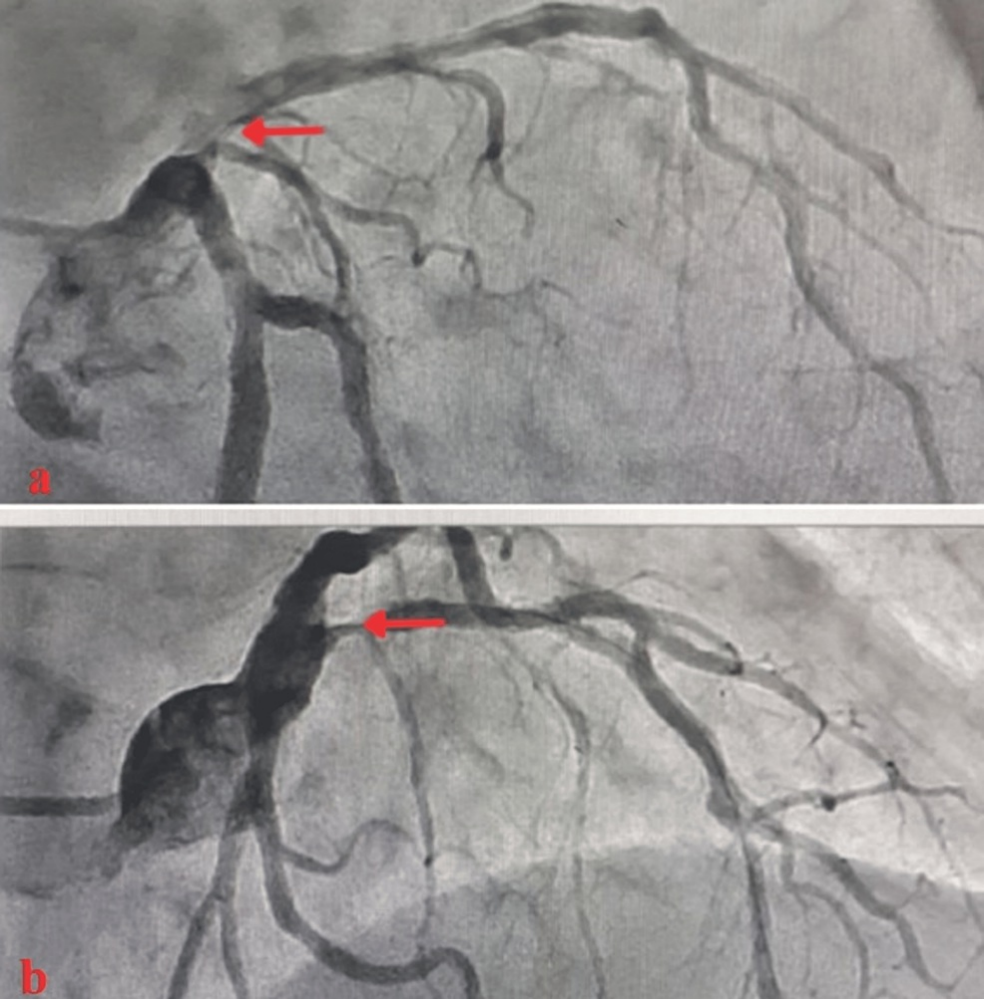
Coronary angiogram (a, AP cranial view; b, LAO cranial view) shows type C lesions in the proximal left anterior descending artery. AP: anterior-posterior; LAO: left anterior oblique

**Figure 2:**
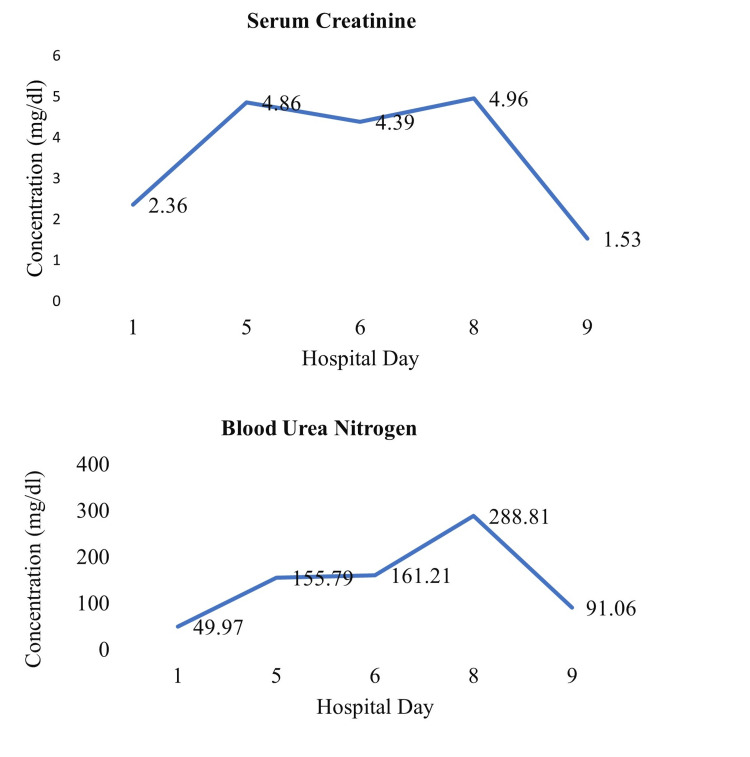
The trend of serum creatinine and blood urea nitrogen (BUN) throughout hospital stay. Normal creatinine value: 0.42-1.06 mg/dl; Normal BUN value 18-45 mg/dl

**Figure 3:**
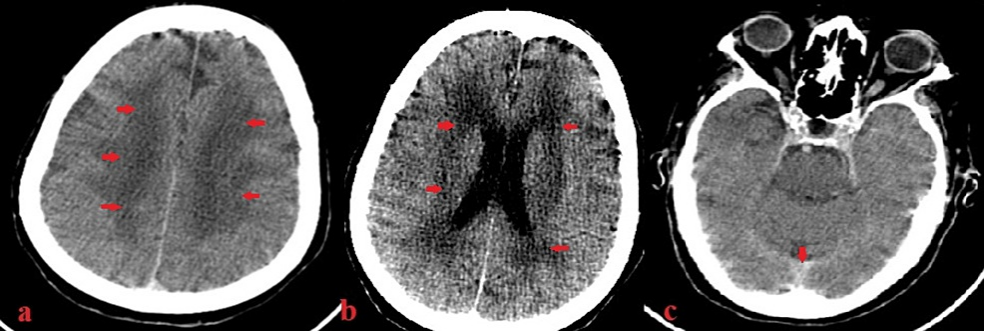
Noncontrast computerized tomography scan axial view: a and b) Shows low attenuation areas (red arrows) in bilateral periventricular region suggestive of microvascular ischemic changes, c) Postetior falx and tentorium cerebelli appear hyperdense (red arrow) suggestive of subarachnoid hemorrhage.

A 75-year-old woman with a past medical history of hypertension presented to the emergency department with sudden-onset chest pain lasting two to three hours. The electrocardiogram (EKG) showed ST-segment elevation in leads V1 to V5, and she was diagnosed with anterior-wall myocardial infarction. Baseline investigations are shown in Table 1. Coronary artery revascularization via the right radial artery revealed proximal 90% of left anterior descending artery (LAD) stenosis with clots (Figure 1). The left circumflex and the right coronary artery (RCA) were non-dominant and normal. These findings were consistent with single-vessel coronary artery disease (SVCAD). Thus, she underwent primary PCI with a single drug-eluting stent placed to LAD through the left main stem of the coronary artery (LMCA). LAD was wired with BM wire and parked distally. Promus Premiere 3.0*38 was deployed proximal to LAD. Adjuvant ballooning was done with a non-compliant Trek 3.5X12. Post-PCI thrombolysis in myocardial infarction (TIMI) grade III was achieved. DAPT including aspiring and P2Y12 inhibitor were started after the PCI procedure. An hour later, she developed drowsiness, confusion, and hemiparesis (motor power 1/5 in all four limbs). In the setting of deranged renal function (Figure 2), a non-contrast CT scan of the brain was performed, which showed hypodensities in the periventricular region bilaterally (Figures 3A, 3B) which reflects ischemic changes. In addition, mild sulcal effacement and hyperdensity were noted in the subarachnoid space without a midline shift (Figure 3C), suggesting SAH. The critical decision regarding continuing dual antiplatelet agents DAPT was pending. Magnetic resonance imaging without contrast was not beneficial and worsening renal function limited obtaining contrast studies such as magnetic resonance angiography. Based on her clinical picture and CT findings, a multidisciplinary team comprising cardiologists, neurologists and radiologists reached a consensus to treat it as a post-PCI white matter stroke. The patient was out of the window for administering the tissue plasminogen activator (tPA). It was decided to continue the DAPT. Gradually, she regained full consciousness and motor power increased to 4/5. A repeat CT scan of the brain confirmed the reversal of the initial changes. She also developed contrast-mediated acute kidney injury with serum creatinine peaking at 4.89 on day 5, returning to 1.53 on discharge. She was eventually discharged on aspirin 75 mg once a day, clopidogrel 75 mg once a day and rosuvastatin 20 mg once a day after nine days of hospital stay.

**Table 1:** Baseline blood investigations on arrival to emergency department. WBC: white blood cells; RBC: red blood cells; Hgb: hemoglobin; HCT: hematocrit; MCV: mean corpuscular volume; MCH: mean corpuscular hemoglobin; MCHC: mean corpuscular hemoglobin concentration

	Day 1	Normal value	Units
WBC	13.9	4-11	x 10^3^/ µL
RBC	4.76	4-6	x 10^6^/ µL
Hgb	13	11.5-17.5	g/dL
HCT	38.1	36-54	%
MCV	80	76-96	fL
MCH	27.2	27-33	pg
MCHC	34	33-35	g/dL
Platelet	298	150-450	x 10^3^/ µL
Neutrophils	70	40-75	%
Lymphocytes	22	20-45	%
Monocytes	6	2-10	%
Eosinophils	2	0-6	%
Sodium (Na)	135	135-150	mmol/L
Potassium (K)	5.21	3.5-5.1	mmol/L
Chloride (Cl)	99.4	96-112	mmol/L
Blood urea	49.97	18-45	mg/dL
Creatinine	2.34	0.42-1.06	mg/dL

## Discussion

This case demonstrates stroke as a possible cause of neurological symptoms following high-risk PCI, especially in the elderly population with impaired renal function.

Stroke is a rare but severe complication after PCI, with an overall incidence of approximately 0.56% [4]. However, individuals predisposed to risk factors including old age, arterial disease, atherosclerotic plaques, atrial fibrillation, and cardiogenic shock, have a significantly higher risk of post-PCI stroke. This case report describes a 75-year-old woman who developed drowsiness, confusion, and hemiparesis an hour after PCI. Thus, the patient's symptoms and neuroimaging findings suggest a white matter stroke, which was initially masquerading as SAH. Furthermore, the patient had a history of hypertension, which is associated with sub-cortical hypodensities of presumed vascular origin [5], thereby predisposing her to the development of ischemic stroke.

Neuroimaging is a useful technique to diagnose contrast retention and rule out post-PCI complications of ischemic or hemorrhagic changes. Due to the patient’s deranged renal function tests and the risk of contrast-induced neurological injury [6], a contrast-enhanced CT scan or brain MRI could not be used to confirm the diagnosis. A post-PCI SAH requires immediate discontinuation of DAPT, thereby increasing the risk of stent thrombosis, whereas the development of post-PCI ischemic stroke requires the use of DAPT. Thus, it is important to differentiate between SAH and white matter stroke, as their management strategies differ significantly. Following PCI, DAPT is indicated to reduce the risk of ischemic changes, including stent thrombosis, perioperative myocardial infarction, ischemic strokes, and cardiovascular deaths. Discontinuation of DAPT may thereby jeopardize the successful outcome of the PCI procedure.

## Conclusions

In conclusion, it is essential to differentiate between ischemic stroke and SAH in the setting of post-PCI, as both conditions have different management and direct consequences for the patient. The decision about DAPT continuation is critical for the patient and relies on a prompt diagnosis. The clinical correlation with the radiology findings cannot be understated. Whenever doubtful, a revised opinion from a multidisciplinary team in the patient's best interest should not be delayed. White matter stroke is rare, but it can pose a severe diagnostic dilemma that requires early detection and prompt treatment.

## References

[1] Banerjee S, Angiolillo DJ, Boden WE (2017). Use of antiplatelet therapy/DAPT for post-PCI patients undergoing noncardiac surgery. J Am Coll Cardiol.

[2] Albakr A, Ishaque N, Aljaafari D, Sairafi SN (2020). Contrast-induced transient neurological symptoms following percutaneous coronary intervention: a case report. Am J Case Rep.

[3] Gollol Raju NS, Joshi D, Daggubati R, Movahed A (2015). Contrast induced neurotoxicity following coronary angiogram with Iohexol in an end stage renal disease patient. World J Clin Cases.

[4] Spindel J, Karmali D, Chen E, Ghafghazi S (2020). Stroke and spinal infarct caused by percutaneous coronary intervention. BMJ Case Rep.

[5] Sargento-Freitas J, Felix-Morais R, Ribeiro J (2014). Different locations but common associations in subcortical hypodensities of presumed vascular origin: cross-sectional study on clinical and neurosonologic correlates. BMC Neurol.

[6] Ho SK, Lee JK, Lai YJ, Lin TC, Liu CW (2016). Differentiating contrast staining after acute ischemic stroke from hemorrhagic transformation during emergency evaluation. Am J Emerg Med.

